# Venous contribution to sodium MRI in the human brain

**DOI:** 10.1002/mrm.27996

**Published:** 2019-09-25

**Authors:** Ian D. Driver, Robert W. Stobbe, Richard G. Wise, Christian Beaulieu

**Affiliations:** ^1^ Cardiff University Brain Research Imaging Centre School of Psychology Cardiff University Cardiff United Kingdom; ^2^ Department of Biomedical Engineering University of Alberta Edmonton Alberta Canada

**Keywords:** ^23^Na MRI, blood, sodium MRI, tissue sodium concentration, veins

## Abstract

**Purpose:**

Sodium MRI shows great promise as a marker for cerebral metabolic dysfunction in stroke, brain tumor, and neurodegenerative pathologies. However, cerebral blood vessels, whose volume and function are perturbed in these pathologies, have elevated sodium concentrations relative to surrounding tissue. This study aims to assess whether this fluid compartment could bias measurements of tissue sodium using MRI.

**Methods:**

Density‐weighted and B_1_ corrected sodium MRI of the brain was acquired in 9 healthy participants at 4.7T. Veins were identified using co‐registered ^1^H $T_{2}^{*}$‐weighted images and venous partial volume estimates were calculated by down‐sampling the finer spatial resolution venous maps from the $T_{2}^{*}$‐weighted images to the coarser spatial resolution of the sodium data. Linear regressions of venous partial volume estimates and sodium signal were performed for regions of interest including just gray matter, just white matter, and all brain tissue.

**Results:**

Linear regression demonstrated a significant venous sodium contribution above the underlying tissue signal. The apparent venous sodium concentrations derived from regression were 65.8 ± 4.5 mM (all brain tissue), 71.0 ± 7.4 mM (gray matter), and 55.0 ± 4.7 mM (white matter).

**Conclusion:**

Although the partial vein linear regression did not yield the expected sodium concentration in blood (~87 mM), likely the result of point spread function smearing, this regression highlights that blood compartments may bias brain tissue sodium signals across neurological conditions where blood volumes may differ.

## 1 INTRODUCTION

In the brain, sodium plays a key role in neuronal action potentials, mediates the transport of metabolic substrates through cell membranes and is involved in osmoregulation and pH regulation.^1^, ^2^ Sodium (^23^Na) MRI shows promise as a marker for cerebral metabolic dysfunction in studying stroke,^3^, ^4^, ^5^, ^6^ brain tumor,^7^, ^8^, ^9^, ^10^, ^11^ and neurodegenerative pathologies.^12^, ^13^, ^14^, ^15^, ^16^, ^17^ However, the ^23^Na MRI signal is greater in cerebrospinal fluid (CSF),^18^, ^19^, ^20^, ^21^ such that measurements of tissue sodium can be biased by tissue atrophy. Signal contamination from CSF can be corrected prospectively, by suppressing CSF signal using an inversion recovery sequence,^22^ or retrospectively, using partial volume correction.^21^ Another cerebral fluid compartment, which has not been considered previously in the context of sodium MRI quantification, is cerebral blood vessels that occupy approximately 5% of the cerebral volume.^23^ Human blood sodium concentrations have been measured in vitro by sodium MR spectroscopy as ~87 mM,^24^, ^25^ with 2 main constituents of blood being ~60% plasma, an extracellular compartment with ~150 mM sodium concentration, and ~40% red blood cells, an intracellular compartment with ~15 mM sodium concentration.^25^ By using a shift reagent in the rat brain, a significant 16% intravascular sodium signal increase was observed with NMR during a hypercapnia‐induced increase in cerebral blood volume (CBV).^26^

The space occupied by cerebral blood vessels is perturbed in pathologies, such as brain tumor, stroke and multiple sclerosis. Angiogenesis is a key part of the pathophysiology in cancer,^27^ leading to a focal increase in CBV. Stroke, whether it is ischemic or hemorrhagic, has a significant effect on CBV.^28^ Multiple sclerosis lesions appear to form around veins,^29^, ^30^ so the sodium MRI signals in these lesions will include a significant venous compartment. Therefore, understanding the scale of sodium signal in cerebral blood, relative to tissue, becomes important in interpreting sodium signals when investigating the pathophysiology of these conditions using sodium MRI, where CBV is likely to be perturbed. In this study, the ^23^Na MRI signal from cerebral veins, which are readily identifiable using $T_{2}^{*}$‐weighted MRI^31^ was assessed and compared with the ^23^Na MRI signal from brain tissue.

## 2 METHODS

**Figure 1:**
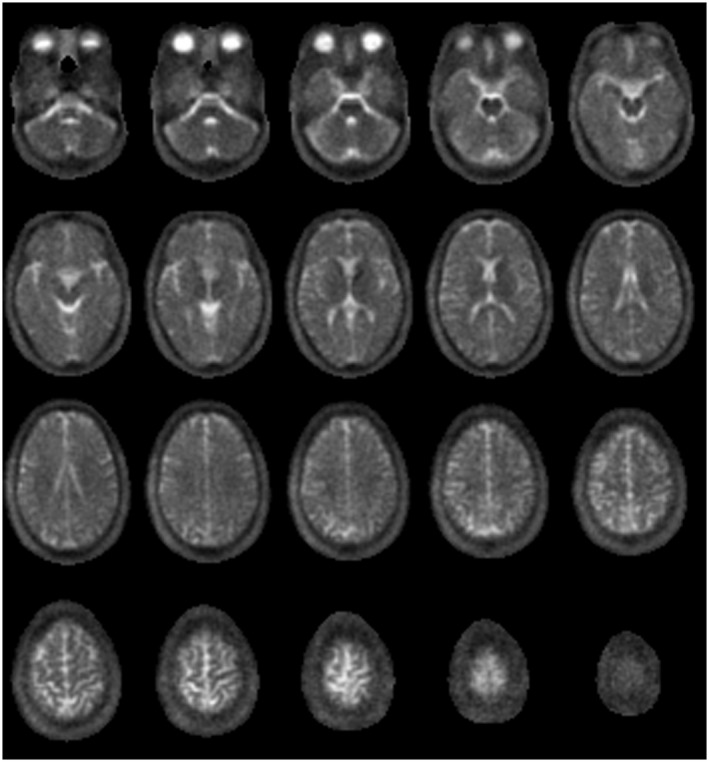
Twenty axial slices of ^23^Na, showing the central portion of the data, from a healthy 30‐year‐old male volunteer

**Figure 2:**
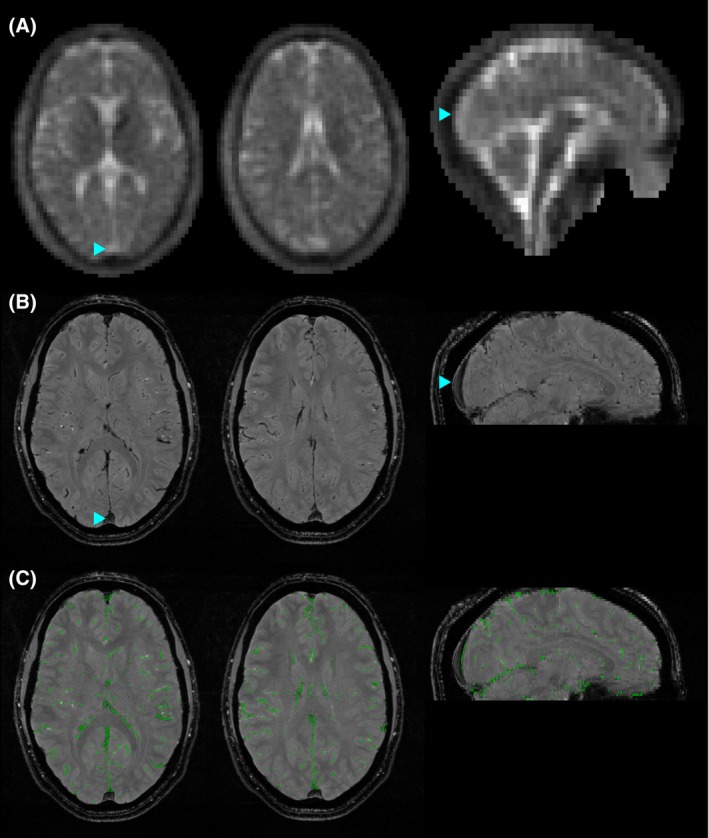
Sodium signal in the superior sagittal sinus (SSS), a major vein, and demonstration of vein masking. A, Two axial slices and 1 sagittal slice of an example density‐weighted ^23^Na dataset, demonstrating higher sodium signal in the SSS (blue arrowhead) than the surrounding tissue. B, ^1^H FLASH image with veins appearing hypointense. Blue arrowheads mark the same point on the SSS as shown in (A). C, Demonstrating the binary vein mask (green) overlaid on the ^1^H FLASH image from which the mask is calculated. Note that the SSS is not included in the mask as its diameter is larger than the 3 mm spatial filter. The images are from a healthy 30‐year‐old male volunteer

Nine healthy subjects (age 30 ± 6 years, 5 female/4 male) participated in this study. The Health Research Ethics Board at the University of Alberta approved this study and subjects gave written informed consent. MRI data were acquired on a Varian Inova 4.7T whole‐body system in 2 contiguous sessions with (i) ^23^Na MRI data with a single‐tuned birdcage head coil and (ii) ^1^H MRI data with a birdcage transmit head coil and 4‐element receive array. Density‐weighted whole‐brain ^23^Na images (Figures 1 and 2A) were acquired using twisted projection imaging with 18 ms readout duration and voxels of 3.2 × 3.2 × 6.4 mm^3^ (defined by 1/(2k_max_)). A total of 6000 projections (*ρ* = 0.2) fully sampled k‐space with sampling density designed for an approximately Hamming filtering shape to reduce CSF ringing.^32^ The parameters of TR = 85 ms, echo time = 0.11 ms, and flip angle = 30° were selected to minimize both T_1_ and rapid biexponential T_2_ weighting as well as signal loss from residual quadrupole splitting.^33^ The acquisition of 1 average yielded a scan time of 8.5 min. B_1_ maps were acquired from 2 low resolution images using a double flip‐angle approach and used to correct the density weighted signal variation as a result of flip angle ($B_{1}^{+}$) and receive sensitivity ($B_{1}^{-}$). Anatomical ^1^H MPRAGE (whole‐brain, 1 mm isotropic, repetition time = 1650 ms, inversion time = 725 ms, echo time = 4.5 ms, flip angle 10°) and FLASH (50 axial slices, 0.5 × 0.5 × 2 mm^3^, repetition time = 1540 ms, echo time = 15 ms) were acquired for segmentation of tissue and veins, respectively.

N4 bias field correction^34^ was performed on the MPRAGE and FLASH images before segmentation. Tissue segmentation was performed on the MPRAGE using the FSL Brain Extraction Tool (BET) and FMRIB’s Automated Segmentation Tool (FSL 5.0.9, fMRIB, Oxford, UK). This resulted in gray matter (GM), white matter (WM), and CSF partial volume estimate (PVE) maps. Segmentation of veins was performed on the FLASH images, because cerebral veins are hypo‐intense on these $T_{2}^{*}$‐weighted images. To minimize image contrast other than signal decrease over the small spatial scales associated with veins, a 3 mm full‐width‐half‐maximum Gaussian high‐pass spatial filter was applied to the FLASH images. A brain mask (performed using BET) eroded by 3 voxels (7 × 7 kernel) was then applied to remove filter artifacts at the edge of the brain. The hypo‐intense veins on the $T_{2}^{*}$ images are manifest with negative values on the high‐pass filtered images, while brain tissue retains an average contrast near zero apart from small positive/negative variation at tissue boundaries. A minimum negative threshold was selected to create a binary vein mask, while ensuring that negative high‐pass values resulting from tissue boundaries did not contribute to the mask (example shown in Figure 2). When the 0.5 × 0.5 × 2 mm^3^ binary vein masks are resampled to the 3.2 × 3.2 × 6.4 mm^3^ voxel size of the ^23^Na images (see below), these masks become vein PVE maps with 131 FLASH voxels for each ^23^Na voxel.

For the purposes of subsequent analysis, ^23^Na images were reconstructed onto an 88 × 88 × 44 grid (3.2 × 3.2 × 6.4 mm^3^). However, solely to assist with registration from the MPRAGE and FLASH images to ^23^Na space, an additional 616 × 616 × 308 reconstruction was also performed, using zero filling. The finer spatial resolution of this reference provided improved registration accuracy, compared with co‐registering the MPRAGE and FLASH directly to the low‐resolution ^23^Na image. Rigid‐body registration was performed using FMRIB's Linear Image Registration Tool (FSL, FMRIB, Oxford, UK) to realign MPRAGE and FLASH images to the zero‐filled ^23^Na image. These registrations were applied to the tissue segmentation maps. The zero‐filled 616 × 616 × 308 grid maps directly onto the low‐resolution 88 × 88 × 44 grid through a set of 7 × 7 × 7 sub‐voxels within each single voxel of the low‐resolution grid. Therefore, the tissue segmentation maps were down‐sampled from the 616 × 616 × 308 matrix to the 88 × 88 × 44 matrix by taking the mean of each 7 × 7 × 7 block of sub‐voxels. This form of downsampling does not cause any additional spatial smoothing (over that arising from the image registration), as no interpolation is required. Note that the FLASH dataset did not cover the most inferior portion of the brain (see Figure 2 for extent), resulting in areas where no venous information was available. Therefore, these regions were excluded from subsequent sodium analysis.

No external references were acquired for calibrating sodium concentration, so signal from vitreous humor (VH) was used as a reference for sodium signals from other tissues. VH volumes in the eyeballs were sufficiently large to provide a central sodium signal free from partial volume bias. A literature value of [Na]_VH_ = 134 mM^20^ was assumed, while the density weighted acquisition parameters mitigated against differences in tissue relaxation values.^33^ A VH region of interest (ROI) was drawn manually on the MPRAGE, then realigned and downsampled to the ^23^Na image. This VH ROI was eroded using a 3 × 3 × 3 cubic voxel structure element and VH sodium signal was measured as the mean across this eroded ROI.

**Figure 3:**
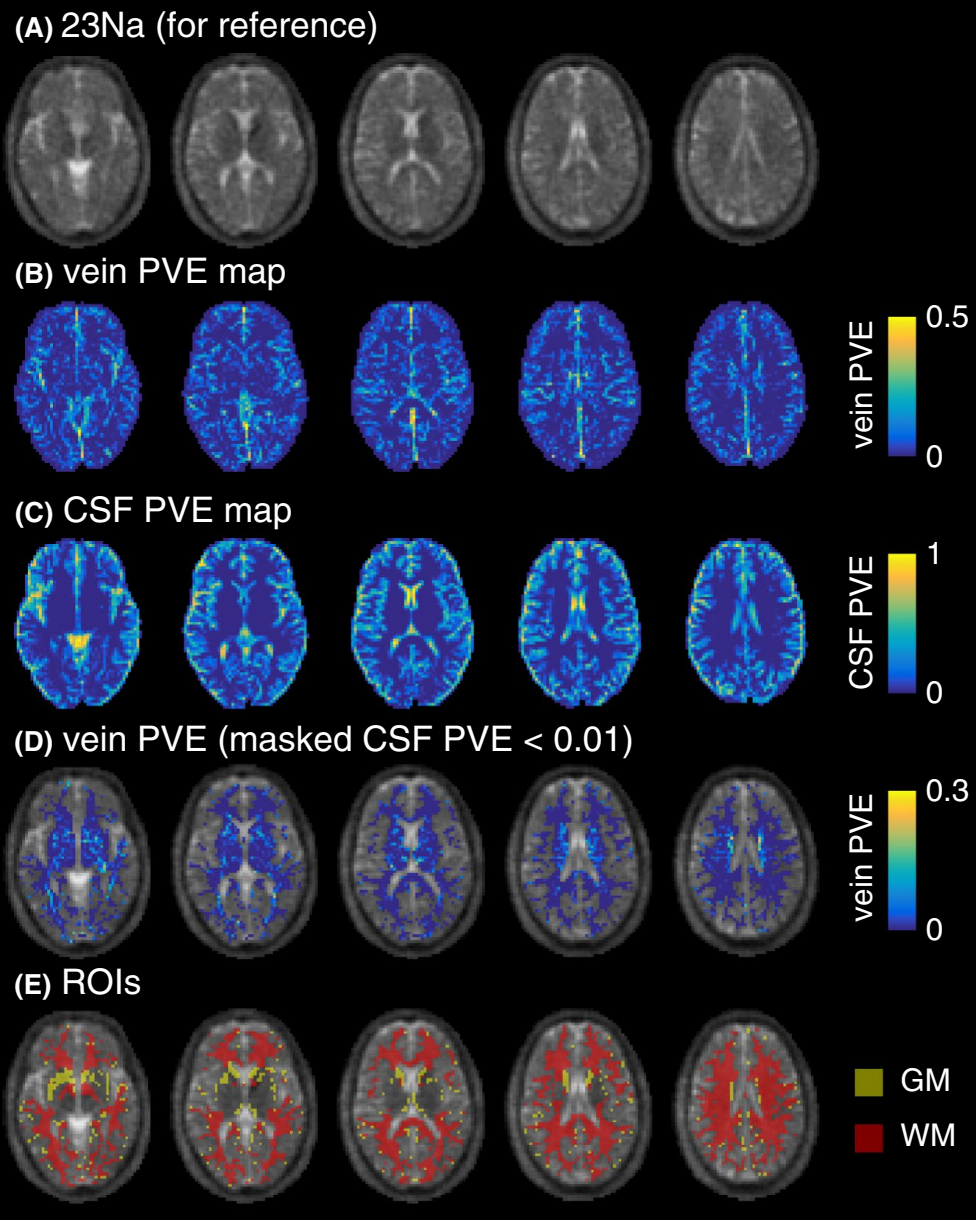
Illustration of partial volume estimate (PVE) maps shown in 5 axial slices from a healthy 30‐year‐old male volunteer. A, ^23^Na image, for reference. B, Vein PVE map. C, CSF PVE map, showing some spatial overlap with vein PVE (N.B. scale not matching (B), optimized for data range). D, Vein PVE map masked by CSF PVE < 0.01, showing the full brain tissue mask used to calculate venous sodium signal (N.B. scale not matching (B, C), optimized for data range). E, Example GM and WM ROIs

A brain tissue mask was created by excluding any voxels with CSF PVE ≥ 0.01. This was necessary to minimize contamination from the relatively high sodium concentration in CSF (~150 mM). Note that larger veins are typically found at the cortical surface and so generally lay adjacent to CSF spaces. The conservative CSF masking procedure eliminates these voxels from analysis. The result of CSF exclusion can be observed by comparing the vein PVE map (Figure 3B) with the CSF excluded vein PVE map (Figure 3D). Within the brain tissue mask, brain tissue was segmented into GM and WM ROIs using a 0.5 PVE threshold. However, the segmentation of the 4.7T MPRAGE images did not perform well in regions of the thalamus and putamen, erroneously classifying parts as WM. These regions were identified manually and excluded from the WM ROIs to ensure proper tissue classification. As a result, these regions were not part of either the WM or GM ROIs; however, they remain within the full brain tissue mask. Representative GM and WM ROIs are shown in Figure 3E.

To determine the effect of elevated sodium concentration within blood on tissue sodium concentration measurements, a linear regression was performed between vein PVE and ^23^Na signal (relative to VH signal) over every voxel within the brain tissue mask. This regression was then redone for voxels only within the WM ROIs and only in the GM ROIs. For each volunteer, this spatial regression yielded ^23^Na blood values (relative to VH) calculated at vein PVE = 1. Note that the superior sagittal sinus (SSS) was not included in the vein PVE map, as it was too large to be resolved by the high‐pass spatial filter during vein segmentation. Alternatively, an ROI including the SSS was drawn manually on the ^1^H FLASH image, and resampled into ^23^Na space creating a SSS PVE map. Average signal was calculated across SSS PVE ≥ 0.5 voxels. These values (converted to concentration according to [Na]_VH_ = 134 mM)^20^ were then compared with tissue sodium values (similarly converted to concentration) directly measured from the WM and GM ROIs, and with blood concentration from the literature. All values were reported as mean ± SEM across 9 subjects. Venous signals were compared with mean tissue signal over the ROI they were calculated from using a Bonferroni‐corrected paired 2‐tailed *t*‐test. SSS signal was compared with GM tissue signal.

## 3 RESULTS

An example of density‐weighted ^23^Na images is shown for the whole brain in Figure 1. Higher sodium signals in veins is most noticeable in the major draining vein of the brain, the SSS (Figure 2A), which is visible in ^23^Na images from all subjects. Veins mapped on the ^1^H FLASH $T_{2}^{*}$‐weighted images occupied 3.1 ± 0.2% of the whole brain volume (mean ± SEM across 9 subjects). The maximum vein PVE within the brain tissue mask ranged from 20‐57% of the voxel (median 36%).

**Figure 4:**
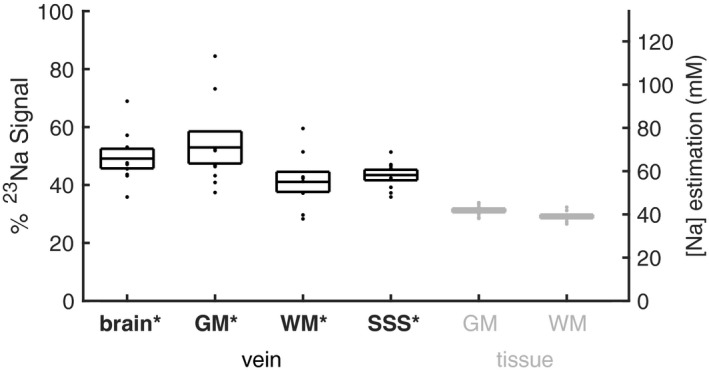
Sodium signals as a percentage of VH sodium signals. Vein sodium signals determined from regression over whole brain tissue, GM, and WM regions and averaged over the SSS are shown in black. Tissue sodium signals from GM and WM are shown in gray. The right‐hand axis shows estimated sodium concentrations, based on a literature value of 134 mM^20^ for VH. Boxes show mean ± SEM across 9 subjects, and dots show each individual subject’s value. *P < 0.05 compared with tissue signal for each region and to GM for the SSS (Bonferroni‐corrected paired *t*‐test)

All vein sodium signal calculations were significantly greater than the mean tissue signal of the ROI they were calculated from, as follows: Vein PVE regression over all voxels within the brain tissue mask yielded a venous sodium signal 49 ± 3% of VH signal, or a VH normalized apparent concentration value of 65.8 ± 4.5 mM (**t(8) = 6.3; *P* = 9 × 10^−4^**). Regression in GM ROIs yielded apparent venous concentration of 71.0 ± 7.4 mM (**t(8) = 4.3; *P* = 0.01**), while regression in WM ROIs yielded apparent venous concentration of 55.0 ± 4.7 mM (**t(8) = 3.5; *P* = 0.03**). SSS venous concentration was measured as 58.2 ± 2.4 (**t(8) = 10.6; *P* = 2 × 10^−5^**). The apparent tissue sodium concentration values measured in the WM and GM ROIs were 39.1 ± 0.8 mM and 41.9 ± 0.9 mM, respectively. Figure 4 presents individual subject data for all measurements.

## 4 DISCUSSION

The regression between vein PVEs and density‐weighted sodium signal yielded average (over all volunteers) apparent sodium concentration in veins of 55 mM‐71 mM (depending on which brain tissue voxels were included in the regression). This is considerably less than the ~87 mM previously measured in vitro for human blood.^24^, ^25^ However, the result that regression produced significantly greater vein concentration values for all brain tissue (69 ± 11%), GM (69 ± 16%), and WM (42 ± 13%) demonstrates that vein partial volume does contribute to the sodium signal within brain voxels. Although this contribution may not be directly visible on a ^23^Na MRI image, it is sufficient to produce the positive correlations measured in this study.

The apparent sodium concentrations reported were calculated by comparing (B_1_ corrected and density‐weighted) sodium signal measurements and regression values with that in VH. Using VH = 134 mM,^20^ apparent sodium concentration values of 39.1 ± 0.8 and 41.9 ± 0.9 mM were measured in WM and GM, respectively. These are at the midpoint of existing literature values.^13^, ^15^, ^16^, ^17^, ^19^, ^20^, ^21^, ^35^, ^36^, ^37^ Although the apparent sodium concentration values reported in this study are sensitive to inter‐subject sodium VH concentration variability, the variance in WM and GM measurements is small across subjects. This can be seen in the standard deviation values reported above and in Figure 4. Although this method of apparent sodium concentration measurement is also sensitive to the accuracy of the VH concentration value from the literature, absolute concentration values are not necessary to demonstrate through regression that sodium signal from veins contributes to brain tissue voxels.

The density‐weighted ^23^Na MRI images have insufficient spatial resolution to directly measure blood sodium signal in any of the intracranial veins, including the SSS. The twisted projection acquisition scheme used for the ^23^Na sequences has a point spread function (PSF) full‐width‐half‐maximum of 7.1 mm in‐plane (and double that out‐of‐plane). This is calculated for the T_2fast_ = 2 ms and T_2slow_ = 17 ms of blood^38^, ^39^ and includes the Hamming‐like filter for Gibbs ringing removal. As a result, the elevated ^23^Na signal originating from veins will be smeared according to the PSF of the sodium images.^40^ Signal originating from a vein will contribute only part of its signal to the voxel in which it resides, the rest will contribute to the surrounding voxels. This PSF smearing (at least partially) explains why the apparent blood sodium concentrations measured from all regression experiments are lower than that expected from previous in vitro study.^24^, ^25^ In addition, the spatial extent of veins will be overestimated due to intra‐voxel dephasing of adjacent tissue signal, a result of the vein’s fringe field. Thus, the vein PVE maps will overestimate the relative contribution of a small vein to the sodium dataset, resulting in the regression of smaller blood sodium concentration values.

The vein PVE–sodium regression including voxels in just WM ROIs yielded smaller apparent blood sodium concentration values than the regressions including voxels in GM. This is likely because the WM ROIs contain primarily small veins. The additional signal contribution to a voxel from small veins will in turn be small, yielding an effect more sensitive to noise. GM contains a greater number of larger veins with greater signal increase.

**Figure 5:**
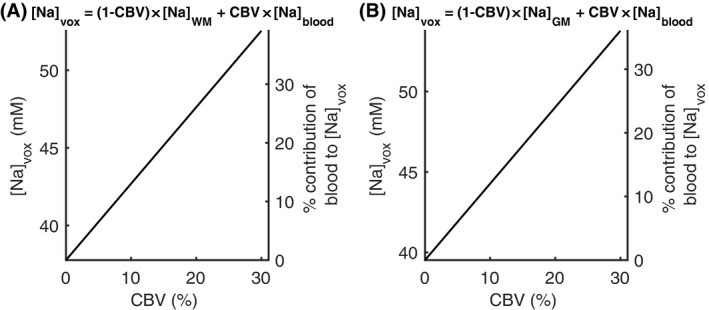
Simulation of the signal contribution from blood to WM (A) and GM (B) tissue sodium concentration measurements. [Na]_vox_ is the sodium concentration that would be measured for [Na]_WM/GM_ containing a CBV with sodium concentration [Na]_blood_ = 87 mM.^24^, ^25^ [Na]_WM_ = 37.8 mM is the WM sodium concentration with the blood contribution removed, calculated based on the measured [Na]_WM_ = 39.1 mM corrected for CBV = 2.7%.^41^, ^42^, ^43^ [Na]_GM_ = 39.5 mM is the GM sodium concentration with the blood contribution removed, calculated based on the measured [Na]_GM_ = 41.9 mM corrected for CBV = 5%.^23^, ^42^, ^43^ The right‐hand axes show the contribution of blood to the total sodium signal. The CBV range of 0 to 30% reflects the typical intra‐subject range of values across voxels in the vein PVE map within the region used to calculate venous sodium signal

To assess the contribution of blood to tissue sodium signals, literature values for CBV of 2.7% for WM^41^, ^42^, ^43^ and 5% for GM^23^, ^42^, ^43^ were assumed, and the sodium signal from all blood vessels was assumed equal to that directly measured in the literature (~87 mM). Correcting for the blood signal using these values reduced the tissue sodium concentration values by 3.4% and 5.7% for WM and GM, respectively. Total sodium concentrations were simulated for a range of CBV values from 0 to 30% (approximate intra‐subject range of vein PVE) in Figure 5. Although the values measured in this study were smeared by the PSF, the full blood vessel signal contribution is still contained within the measured tissue sodium signal, spread across a greater number of pixels.

While this study focuses on cerebral veins (which are visible on the $T_{2}^{*}$‐weighted ^1^H images), the partial volume contribution of arteries is also expected to increase tissue sodium concentration (and likely affected the regression performed in this study). Because the plasma volume fraction is similar in arteries and veins,^44^ these blood vessels should contain similar sodium concentrations. However, the plasma volume fraction is ~25% higher in microvasculature.^23^, ^45^ With plasma sodium concentration on the order of 150 mM,^25^ microvasculature is likely to have a greater sodium concentration than veins and arteries.

The distinct sodium MRI characteristics of blood have significance for studies where the pathophysiology includes cerebral blood vessels, such as brain tumor,^7^, ^8^, ^9^, ^10^, ^11^ stroke,^3^, ^4^, ^5^, ^6^ and multiple sclerosis.^13^, ^15^, ^16^, ^17^ There can be significant focal increases in the size of the blood volume compartment in these pathologies. These results are also of significance for recent studies demonstrating sodium fMRI contrast,^46^, ^47^ where functional responses of interest will be accompanied by focal vasodilation. For example, focal arterial blood volume can increase in the range of 30‐60% in response to a functional stimulus.^48^, ^49^, ^50^, ^51^, ^52^ The apparent tissue sodium signal increase due to an increase in volume of blood vessels could be misattributed to a neuronal mechanism, if not accounted for. Studies of tissue sodium concentration in conditions such as above, where cerebral blood vessels have a significant role, could also assess CBV^41^, ^42^, ^43^, ^53^ to control for the blood volume contribution to the sodium signal.

## 5 CONCLUSIONS

It was demonstrated that tissue sodium concentration increases in proportion to the partial vein contribution to a voxel. Thus, tissue sodium concentrations may be overestimated in the presence of large blood vessels, and may vary as a result of blood vessel and blood flow perturbation.
